# Importance of Cation Species during Sulfate Resistance Tests for Alkali-Activated FA/GGBFS Blended Mortars

**DOI:** 10.3390/ma12213547

**Published:** 2019-10-29

**Authors:** Youngkeun Cho, Joo Hyung Kim, Sanghwa Jung, Yoonseok Chung, Yeonung Jeong

**Affiliations:** 1 Jeonnam and Jeju Branch, Korea Conformity Laboratories (KCL), 64, Oemori-gil, Yeosu-si, Jellanam-do 59631, Korea; young@kcl.re.kr; 2 Construction Technology Research Center, Korea Conformity Laboratories (KCL), 199, Gasan Digital 1-ro, Geumcheon-gu, Seoul 08503, Korea; kjhmole@kcl.re.kr (J.H.K.); yschung24@kcl.re.kr (Y.C.); 3 Yeongnam Division, Korea Conformity Laboratories (KCL), 36, Technosunhwan-ro 12-gil, Yuga-eup, Dalseong-gun, Daegu 42994, Korea; jsh2593@kcl.re.kr

**Keywords:** alkali-activated materials, fly ash, ground granulated blast-furnace slag, sulfate resistance, cation accompanying sulfate

## Abstract

In this study, the changes in mass, compressive strength, and length of blended mortars were analyzed to investigate their sulfate resistance according to the ground granulated blast furnace slag (GGBFS) blending ratio and type of sulfate solution applied. All alkali-activated mortars showed an excellent sulfate resistance when immersed in a sodium sulfate (Na_2_SO_4_) solution. However, when immersed in a magnesium sulfate (MgSO_4_) solution, different sulfate resistance results were obtained depending on the presence of GGBFS. The alkali-activated GGBFS blended mortars showed a tendency to increase in mass and length and decrease in compressive strength when immersed in a magnesium sulfate solution, whereas the alkali-activated FA mortars did not show any significant difference depending on the types of sulfate solution applied. The deterioration of alkali-activated GGBFS blended mortars in the immersion of a magnesium sulfate solution was confirmed through the decomposition of C–S–H, which is the reaction product from magnesium ions, and the formation of gypsum (CaSO_4_·2H_2_O) and brucite (Mg(OH)_2_).

## 1. Introduction

With the increased awareness of global climate change as a social issue beginning in the 2000s, an increasing need has developed for new materials that can replace ordinary Portland cement (OPC) and thereby reduce the amount of CO_2_ emissions during the cement manufacturing process [1,2,3,4]. It is worth noting that quantified sustainability indexes have been developed for cementitious materials, such as empathetic added sustainability index (EASI) [5]. Some studies [4,6,7,8,9,10,11] have proposed the use of calcium sulfoaluminate (CSA) cement to reduce the firing temperature and total CaO content, resulting in fewer CO_2_ emissions during production as compared to OPC. By contrast, alkali-activated materials (AAMs) have also been considered as a candidate to replace OPC because AAMs can be manufactured through the recycling of industrial by-products, such as ground granulated blast-furnace slag (GGBFS) and fly ash (FA) [12,13,14,15,16,17,18]. 

In general, AAMs can be divided into two types depending on the reaction products: (1) a calcium-rich binder such as alkali-activated GGBFS, producing a calcium (alumino-)silicate hydrate [C-(A)-S-H] similar to an OPC hydrate, and (2) a low calcium binder (also called a geopolymer), such as alkali-activated metakaolin and/or class F FA, mainly producing amorphous alkali aluminosilicates gels [alkali-A-S(-H)], which are also regarded as a phase resembling that of nano-scale zeolite [4,19,20,21,22]. Although numerous chemicals such as caustic alkalis (MOH), both non-silicates (M_2_CO_3_, M_3_PO_4_, and M_2_SO_4_) and silicates (M_2_O·nSiO_2_), alkaline earth oxides (CaO and MgO), and alkaline earth hydroxides (Ca(OH)_2_, Ba(OH)_2_, and Ba(OH)_2_·8H_2_O) have been used as activators, NaOH, Na_2_CO_3_, Na_2_O·nSiO_2_, and Na_2_SO_4_ are the most widely available chemicals [12,18,23,24,25,26]. Some potassium compounds and/or blended alkali compounds including potassium chemicals have been used in laboratory studies and special applications such as structural plaster [27], but their potential applications are slightly limited owing to their high costs [23].

Such alternative materials for use as concrete binders should possess sufficient durability, particularly a resistance to sulfate [28,29]. The deterioration of concrete exposed to sulfate salts is the result of the diffusion of sulfate through the pores, generating stresses from the formation of expansive reaction products and the mechanical response of the entire material owing to stresses such as spalling and cracking [30]. In general, the deterioration mechanism occurring through a sulfate attack can be explained by the following two processes. First, as shown in Equations (1) and (2), the Ca(OH)_2_ in concrete reacts with sulfate ions (SO_4_^2−^) to form gypsum (CaSO_4_·2H_2_O), causing an expansion. Second, the reaction of gypsum with calcium aluminate hydrate, as described in Equation (3), causes a degradation of the expansion and delamination, among other properties, owing to the formation of ettringite [31,32].

Na_2_SO_4_ + Ca(OH)_2_ + 2H_2_O → CaSO_4_·2H_2_O + 2NaOH (1)

MgSO_4_ + Ca(OH)_2_ + 2H_2_O → CaSO_4_·2H_2_O + Mg(OH)_2_ (2)

3CaO·Al_2_O_3_·12H_2_O + 3(CaSO_4_·2H_2_O) + 14H_2_O → 3CaO·Al_2_O_3_·3CaSO_4_·32H_2_O (3)

The calcium hydroxide and C–S–H in hydrated Portland cement paste are converted into gypsum through a sulfate attack depending on the cationic type (i.e., Na^+^, K^+^, or Mg^2+^) in the sulfate solution [33]. Deterioration by magnesium sulfate converts the calcium hydroxide into gypsum, and magnesium hydroxide is formed simultaneously. Owing to the extremely low solubility of the magnesium hydroxide, the pH of the saturated solution reaches approximately 10.5, which causes a decomposition of C–S–H and a liberation of the lime. The lime then reacts with magnesium sulfate to produce additional magnesium hydroxide and gypsum. Moreover, the magnesium hydroxide reacts with the silicate hydrate produced through the decomposition of C–S–H to produce magnesium silicate hydrate, which does not have cohesive properties, unlike C–S–H. These continuous chemical reactions cause a significant reduction in the compressive strength [34].

The effects of an external sulfate attack by a 5% Na_2_SO_4_ solution on the mechanical and microstructural properties of alkali-activated slag (AAS) have been studied in comparison with those of Portland‒slag cement [35]. As a result, the sulfate resistance of AAS at 90 days was shown to be remarkably better than that of Portland‒slag cement, which is attributed to the absence of portlandite [Ca(OH)_2_]. These experimental results are consistent with those of a comparative study on the compressive strength of AAS and OPC exposed to a 5% magnesium sulfate and 5% sodium sulfate solution for one year. The results indicate that the compressive strength of OPC concrete decreases by 25–37%, whereas the compressive strength of AAS decreases by 17–23% [36]. Bakharev evaluated the sulfate resistance of geopolymers cured for 24 h at 95 °C using three alkali activators (NaOH, Na_2_SiO_3_, and NaOH + KOH). It was found that the best resistance to sulfate was a NaOH-activated geopolymer, and this excellent resistance is due to the small average pore size of the samples prepared with a NaOH activator. In addition, the compressive strength of the specimens increases by 4%–12% as the exposure time increases [37]. Bhutta et al. also reported that the compressive strength of OPC concrete decreases by 63% after one year of immersion in a 5% sodium sulfate solution, whereas that of geopolymer concrete increases by 7% [38]. The reason for the increase in compressive strength is that sodium sulfate acts as an activator, not as an aggressive agent, and the alkali activation continues in the sulfate solution [39,40]. After evaluating the sulfate resistance using sodium and magnesium sulfate on an alkali-activated fly ash/slag (at a 1:1 ratio), Ismail et al. [39] reported that a sulfate attack is highly dependent on the nature of the cations associated with the sulfate rather than the sulfate itself. An alkali-activated binder continues to stabilize in a sodium sulfate solution by acting as an activator, whereas it causes a loss of structural and dimensional integrity in a magnesium sulfate solution through the formation of gypsum. Although many studies were carried out on the sulfate-induced deterioration of alkali-activated materials, the relationship between the property deterioration of the materials and experimental conditions such as material variables and sulfate conditions should be studied more. Arbi et al. [30] also reported that the relationship between the compressive strength and sulfate treatment was still unclear. 

This study evaluated the sulfate resistance of an alkali-activated FA-based geopolymer and GGBFS blended mortars according to the types and concentrations of the sulfate solution, the GGBFS/FA ratio, the varying initial curing temperature, and the SiO_2_/Na_2_O molar ratio of the activator. Specifically, the sulfate resistance was evaluated by changing the mass, compressive strength, and length of the alkali-activated mortars. Furthermore, an X-ray diffraction analysis was conducted to investigate the change in the mineral composition of an alkali-activated paste immersed in a sulfate solution. 

## 2. Experiments

### 2.1. Materials

FA and GGBFS were used as the source materials during the alkali activation. The chemical compositions and physical properties of the FA and GGBFS are listed in Table 1. The FA is classified as class F fly ash according to ASTM C 618 [41] because SiO_2_ + Al_2_O_3_ + Fe_2_O_3_ is 85.1% and CaO is 3.8%. GGBFS shows 43.0% CaO, 34.3% SiO_2_, and 14.2% Al_2_O_3_. The Blaine surface area is 381 m^2^/kg for FA and 428 m^2^/kg for GGBFS, and the density is 2210 kg/m^3^ for FA and 2890 kg/m^3^ for GGBFS. A fine aggregate is used in accordance with ISO 679. To prepare the activator solutions, a sodium silicate solution, i.e., a water glass (28.2% SiO_2_, 9.3% Na_2_O, and 62.5% H_2_O) and pure sodium hydroxide are adopted. Sulfate solutions were prepared using magnesium sulfate (MgSO_4_) and sodium sulfate (Na_2_SO_4_) reagents.

**Figure 1:**
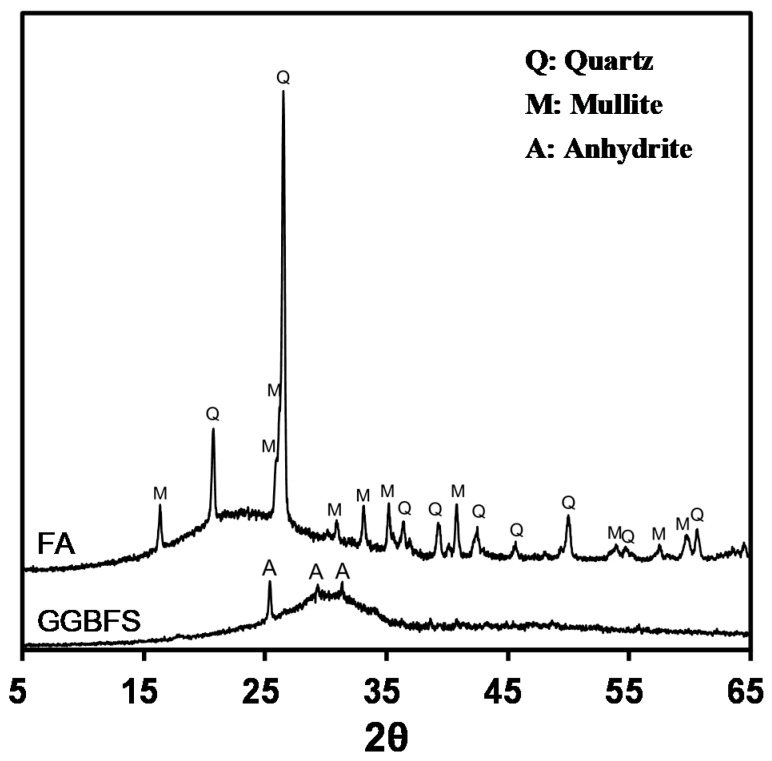
X-ray diffraction patterns of FA and GGBFS.

The X-ray diffraction patterns of raw FA and GGBFS are shown in Figure 1. The major crystalline components in FA are quartz (SiO_2_) and mullite (3Al_2_O_3_·2SiO_2_), which have been commonly identified in FA [39,42,43,44]. For GGBFS, an amorphous phase of 95% or higher occurs because of the precipitation during rapid cooling when completely melted during the steel-making process. Therefore, only the anhydrite (CaSO_4_) added during the blast furnace slag grinding process [43,45,46] was confirmed to be a crystalline component. Both FA and GGBFS show a hump as an amorphous phase characteristic in their X-ray diffraction patterns, and an amorphous hump was located between 15° and 35° 2θ.

### 2.2. Mix Proportions

In a mortar mixture design, the mass ratio of the binder to fine aggregate was 1:3, the water/binder ratio was 0.5, and ISO standard sand (ISO 679) was used as the fine aggregate [47]. The detailed procedure for obtaining the optimum amount of activator and extra water is as follows: (1) determine the molar ratios of SiO_2_/Na_2_O, i.e., Ms, (2) calculate the amount of sodium silicate solution to be added from the determined Ms, (3) calculate the amount of Na_2_O by converting the required Na_2_O into NaOH, except for the amount of Na_2_O in the sodium silicate solution, and (4) add water to make a water/binder ratio of 0.5 including the amount of H_2_O in the sodium silicate solution. The GGBFS blended mortar was cured at room temperature (23 ± 2 °C), whereas the FA-based geopolymer mortar was cured at 70 °C because it demonstrates no strength development when cured at room temperature. Therefore, mortar mixed with an FA-based geopolymer and GGBFS was cured at both 23 °C and 70 °C for an evaluation of the sulfate resistance according to the curing temperature. To evaluate the effect of Ms (SiO_2_/Na_2_O molar ratio) on the sulfate resistance, the Ms values were adjusted to 1.0, 1.5, and 2.0 when 50% GGBFS was mixed (S50).

The overall mixture design used in this study is summarized in Table 2. The activator concentration was 8% Na_2_O with an Ms of 1.4 in the FA-based geopolymer and 4% Na_2_O with an Ms of 1.0, 1.5, and 2.0 in the GGBFS blended mortars. It should be noted that the Na_2_O content and Ms value of the alkaline activators are crucial factors in the property development of the alkali-activated materials [48]. For each case, GGBFS was replaced with 30 wt.% and 50 wt.% of the FA amount, respectively. The mortar specimen preparation method given in ISO 679 was followed for mixing and placement [47]. After mixing, the FA-based geopolymer specimens were cured at 70 °C, and the GGBFS blended specimens were cured at both 23 °C and 70 °C. High-temperature curing, i.e., at 70 °C, was carried out in a curing chamber, and the surface of the mortar was sealed with a polypropylene film to prevent evaporation of water during the curing process. After 24 h, the specimens were demolded and maintained at a constant temperature and relative humidity (RH) until the end of the test periods (at 23 °C and 60% RH). The size of the specimen used for testing the changes in compressive strength and mass was 40 mm × 40 mm × 160 mm, whereas the size for testing the change in length was 25.4 mm × 25.4 mm × 295 mm. To investigate the mineralogical compositions, cement paste specimens (20 mm × 20 mm × 20 mm) were prepared and cured for up to 28 days and then immersed in a 10% sulfate solution to collect the samples based on age. Similar to the mortar mixture design, the activator concentration was 8% Na_2_O with an Ms of 1.4 in the FA-based geopolymer and 4% Na_2_O with an Ms of 2.0 in the GGBFS blended paste (S30). The water-to-binder ratio (w/b) of the paste was fixed at 0.35. The specimens were immersed in isopropanol for 24 h to remove water in the hardened paste. The samples were then vacuum-dried at 40 °C for 24 h, pulverized to 75 μm or less, and analyzed through X-ray diffraction [45,46,49].

### 2.3. Test Methods

A 10% sulfate solution was prepared by dissolving 100 g of magnesium sulfate or sodium sulfate in 800 g of water, and adding distilled water to make 1 L of total solution. Sulfate immersion experiments were carried out by placing small pieces of wood at the bottom of a plastic box and placing the specimens on top. The sulfate solutions were replaced every two months to maintain their concentration. The plastic boxes on which the specimens were placed were stored in a curing room and maintained at a constant temperature (23 ± 2 °C) and humidity (60% of RH). The change in mass was measured by removing the water on the surface of the test specimen immersed in the sulfate solution based on age. The change in length was measured by inserting studs at both ends of the specimen and normalized to the length of the specimen cured at 28 days as a reference length. Note that the length changes in different concentrations of magnesium sulfate solution from 2.5% to 10% were additionally investigated. The length of the specimen immersed in the sulfate solution was measured according to KS F 2424 [50] based on age and calculated using Equation (4).

(4) $$\mathrm{Length}\;\mathrm{change}\;\left(\%\right)=\frac{L_{t}-L_{i}}{L_{i}}\times 100$$

where *L*_t_ is the length (mm) on the immersion day (t), and *L*_i_ is the length (mm) before immersion (on day 28).

The compressive strength of the specimen was measured according to ISO 679, and the loading rate of the compressive strength tests was 2400 ± 200 N/s. XRD experiments were carried out using a MiniFlex 600 diffractometer, manufactured by Rigaku corporation, Tokyo, Japan, (40 kV and 20 mA) with a scanning range of 5° to 65° 2θ, using a step size of 0.02, at 2 s/step. Note that Cu-Kα radiation (λ = 1.5418) was used for the XRD experiments.

## 3. Results and Discussion

### 3.1. Change in Mass

To evaluate the sulfate resistance according to the amount of GGBFS in the alkali-activated binder, the amounts of GGBFS were adjusted to 0%, 30%, and 50%, as shown in Table 2. The initial curing regimes of FA100 were 24 h at 70°C, whereas those of S30 and S50 were 24 h at 23 °C and 70 °C, respectively. After the initial curing, all specimens were cured in a chamber at 23 °C and 60% relative humidity for 27 days, and then immersed in a 10% sodium sulfate (Na_2_SO_4_) solution or a 10% magnesium sulfate (MgSO_4_) solution. The change in mass of the specimen was measured at each test age.

**Figure 2:**
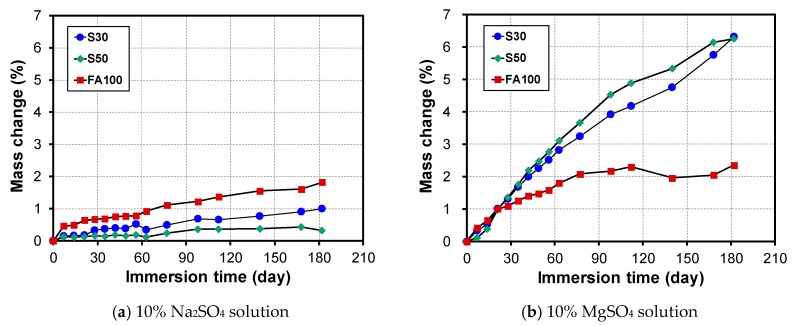
Change in mass depending on GGBFS content, immersed in (**a**) 10% Na_2_SO_4_ solution and (**b**) 10% MgSO_4_ solution.

As shown in Figure 2, the difference in the change in mass based on the immersion time is significant depending on the type of sulfate solution applied. For immersion in the 10% Na_2_SO_4_ solution, FA100 showed the largest mass increase of 1.8% at 181 immersion days, and the change in mass tends to decrease as the amount of GGBFS increases.

**Figure 3:**
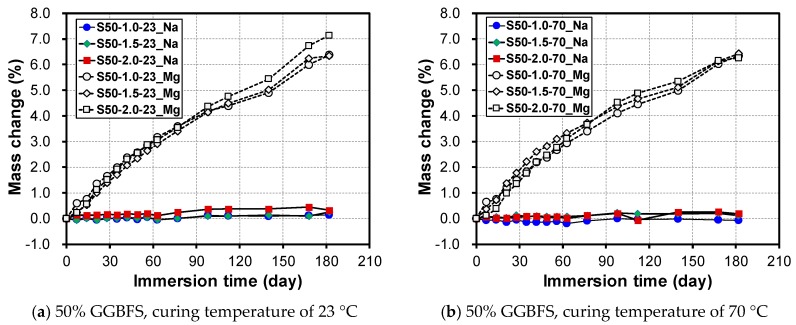
Change in mass by sulfate solution type, Ms, and initial curing temperature at (**a**) 23 °C and (**b**) 70 °C.

For the immersion in a 10% MgSO_4_ solution, the increase in mass of FA100 at 181 days of immersion was 2.3%, which is similar to that of the 10% Na_2_SO_4_ solution. However, for S30 and S50, the mass increased continuously as the immersion increased in the 10% MgSO_4_ solution, with increasing rates of 6.3% for S30 and 6.25% for S50 at 181 days. Therefore, this confirms that the sulfate resistance of the alkali-activated GGBFS blended mortar showed an excellent performance for the sodium sulfate solution, whereas the sulfate resistance to the magnesium sulfate solution was poor. It was worth noting that the trends of FA/GGBFS blended mortar were also identical depending on initial curing temperature. The results of S30 and S50 series initially cured at 70 °C are presented in Figure S1 and Figure 3, respectively. 

As is well known, the Ms in the alkali-activated mortars has a significant influence on the microstructure and compressive strength [51,52,53]. In this study, the change in mass was measured based on the duration of immersion by changing the Ms (i.e., to 1.0, 1.5, and 2.0) and curing temperature (23 °C and 70 °C) for S50. As shown in Figure 3, even when the immersion period is increased, the change in mass is extremely small at within 0.4% regardless of the initial curing temperature and change in Ms when immersed in a 10% Na_2_SO_4_ solution. For immersion in a 10% MgSO_4_ solution, both the initial curing temperatures of 23 °C and 70 °C showed a large increase in mass according to the immersion time. At an initial curing temperature of 23 °C, the mass increase of S50-1.0-23_Mg was 6.37%, whereas that of S50-1.5-23_Mg and S50-2.0-23_Mg was 6.33% and 7.14% at 182 days, respectively. However, no difference in the change in mass owing to the initial curing temperature condition was shown.

In summary, the sulfate resistance of alkali-activated FA based geopolymer mortar shows a good performance regardless of the sulfate type, but the sulfate resistance of an alkali-activated GGBFS blended mortar has a greater influence on the type of sulfate solution than the Ms and initial curing temperature. This mass increase is caused by the formation of gypsum through the decomposition of C–S–H by magnesium cations [34].

### 3.2. Change in Compressive Strength

**Figure 4:**
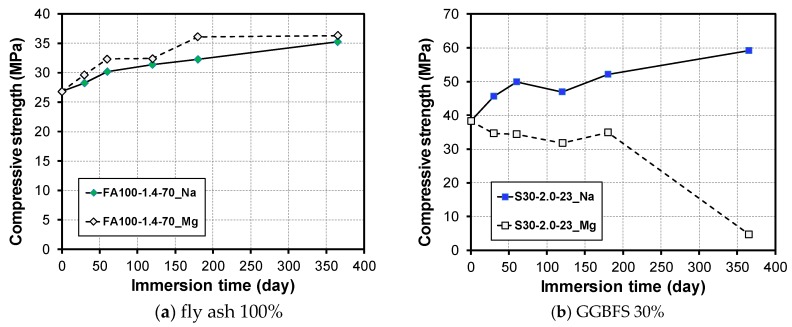

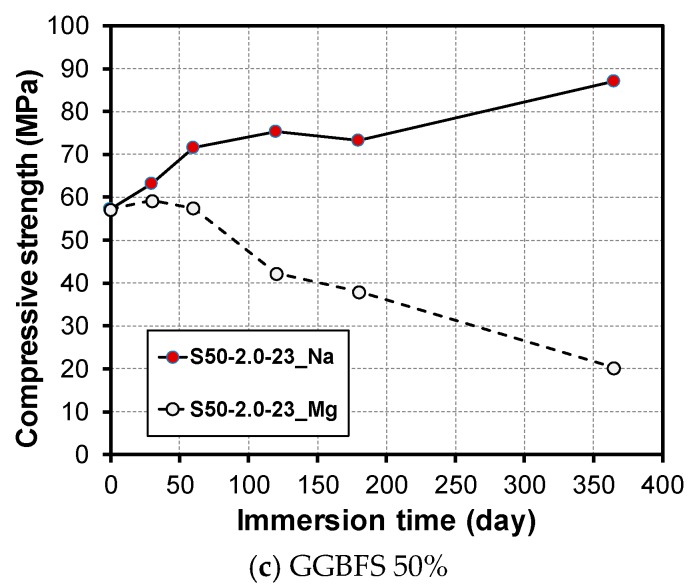
Change in compressive strength based on GGBFS content and sulfate solution type; (**a**) FA100-1.4, (**b**) S30-2.0, and (**c**) S50-2.0 samples.

Figure 4 shows the compressive strength development depending on the GGBFS content (0%, 30%, and 50%) and the types of sulfate solutions (10% Na_2_SO_4_ and 10% MgSO_4_). After the specimens cured for up to 28 days were immersed in a 10% Na_2_SO_4_ solution for one year, the compressive strength of the mortar was found to be higher than the strength before the immersion in all specimens. Similar to the experiment results of the change in mass, it was confirmed that a deterioration of the sulfate did not occur in the 10% Na_2_SO_4_ solution regardless of whether the GGBFS was used.

For immersion in a 10% MgSO_4_ solution, the FA100 exhibited an enhanced compressive strength of 36.3 MPa after a year immersion compared to before the immersion (26.8 MPa). However, when S30 and S50 mixed with GGBFS were immersed in a 10% MgSO_4_ solution for one year, the compressive strength was significantly reduced to 12.3% (4.7 MPa) and 35.4% (20.2 MPa) of the pre-immersion strength. 

As the experiment results of the changes in mass and compressive strength indicate, the FA-based geopolymer mortars were unaffected by the types of sulfate solution applied. The experiment results when using a sodium sulfate solution showed that the compressive strength increases with the immersion time, which is in good agreement with previous studies [37,38,39]. However, when GGBFS is blended, deterioration occurs only during the immersion of the 10% MgSO_4_ solution. This is because Mg(OH)_2_ and CaSO_4_ are formed through the penetration of magnesium cations and sulfate anions, as shown in Equation (2), and thus the mass increases and the compressive strength decreases.

**Figure 5:**
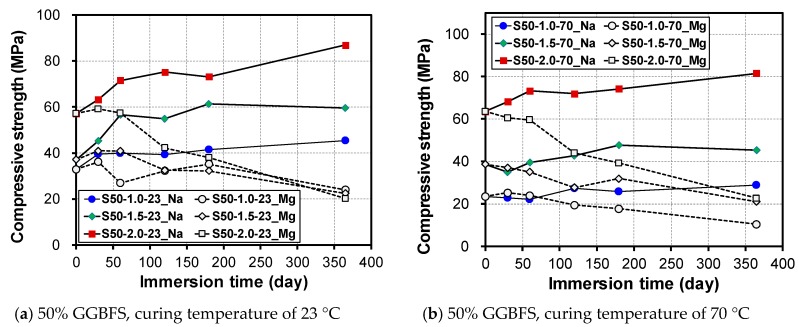
Change in compressive strength based on MS, sulfate solution type, and initial curing temperature at (**a**) 23 °C and (**b**) 70 °C.

Ismail et al. showed that the response of alkali-activated GGBFS-FA blends to a sulfate attack is strongly dependent on the nature of the cation accompanying the sulfate; that is, Na_2_SO_4_ immersion causes little damage, whereas MgSO_4_ causes a loss of structural and dimensional integrity [39]. Figure 5 shows the change in compressive strength of GGBFS blended mortars with respect to Ms and the initial curing temperature. Note that the results of S30 samples initially cured at 70 °C are presented in Figure S2, indicating that the trend is identical to that of S50 samples. For immersion in a 10% Na_2_SO_4_ solution, the compressive strength was increased in all specimens regardless of the change in Ms. The rate of increase of strength in the initial curing specimen at 23 °C was higher than that of the initial curing specimen at 70 °C. The increasing rates of S50-1.0, S50-1.5, and S50-2.0 samples initially cured at 23 °C were 37.6%, 60.4%, and 52.1% while those at 70 °C were 22.2%, 17.1%, and 28.3%, respectively. Similar to the results of the change in mass experiment, there was no decrease in the compressive strength when immersed in a 10% Na_2_SO_4_ solution regardless of the initial curing temperature and change in Ms. For immersion in the 10% MgSO_4_ solution, the compressive strength decreases consistently with the immersion period.

### 3.3. Change in Length 

**Figure 6:**
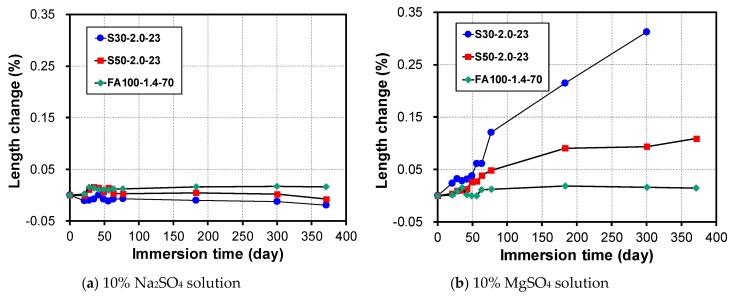
Change in length depending on binder types, immersed in (**a**) 10% Na_2_SO_4_ solution and (**b**) 10% MgSO_4_ solution.

The experiment results on the change in length of an alkali-activated mortar depending on the amount of GGBFS and the type of sulfate solution are shown in Figure 6. As Figure 6a indicates, when immersed in a 10% Na_2_SO_4_ solution for a year, the change in length of S30-2.0-23 was −0.019%, whereas that of S50-2.0-23 and FA100-1.4-70 was −0.007% and 0.016%, respectively. This is an extremely small change compared to the expansion of 0.15% when the OPC mortar is mixed using a water/binder ratio of 0.48 and at a mass ratio of cement to fine aggregate of 1:2.75 when immersed in a 5% Na_2_SO_4_ solution [54]. This implies that sulfate deterioration does not occur even when alkali-activated mortars are immersed in a sodium sulfate solution, similar to the results of the changes in the measured mass and compressive strength. However, when immersed in a 10% MgSO_4_ solution, different changes in length were observed depending on the GGBFS mixing proportions. As shown in Figure 6b, when immersed in a 10% Na_2_SO_4_ solution, the change in length of FA100-1.4-70 was 0.014% at 365 days, whereas that of S50-2.0-23 was 0.110% at 365 days and 0.310% at 300 days, respectively. Therefore, a relatively large amount of sulfate deterioration occurs when alkali-activated mortars mixed with GGBFS are immersed in a magnesium sulfate solution. 

**Figure 7:**
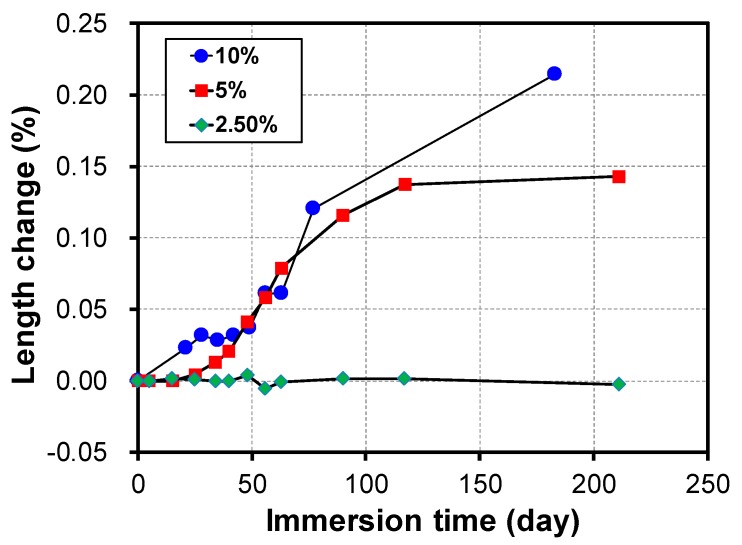
Change in length depending on the concentration of the magnesium sulfate solution.

The alkali activated mortar (S30-2.0-23) was adjusted to 2.5%, 5%, and 10% of the MgSO_4_ concentration to measure the rate of change in the length depending on the immersion time. As shown in Figure 7, it can be seen that the rate of change in the length varies according to the MgSO_4_ concentrations. The maximum rate of change in the length was 0.21% at a MgSO_4_ concentration of 10% at 180 days of immersion, whereas almost no expansion occurred at a MgSO_4_ concentration of 2.5%. Therefore, it can be seen that the deterioration that occurs owing to the presence of sulfate is significantly reduced in the MgSO_4_ solution with a low concentration.

**Figure 8:**
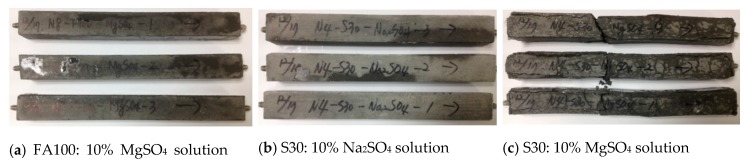
Mortar specimens according to sulfate solution type; (**a**) FA100 samples immersed in 10% MgSO_4_ solution, (**b**) S30 samples immersed in 10% Na_2_SO_4_ solution, and (**c**) S30 samples immersed in 10% MgSO_4_ solution.

Figure 8 shows the specimens of alkali-activated mortar immersed in the sulfate solutions. As the images in Figure 8c indicate, the specimen collapsed owing to an expansion of the mortar when immersed in the 10% MgSO_4_ solution. This is because gypsum is formed through Equation (2) when the alkali-activated mortar is immersed in a 10% MgSO_4_ solution, and the formation of gypsum leads to increases in mass and length and a decrease in the compressive strength.

### 3.4. Changes in Mineral Composition 

**Figure 9:**
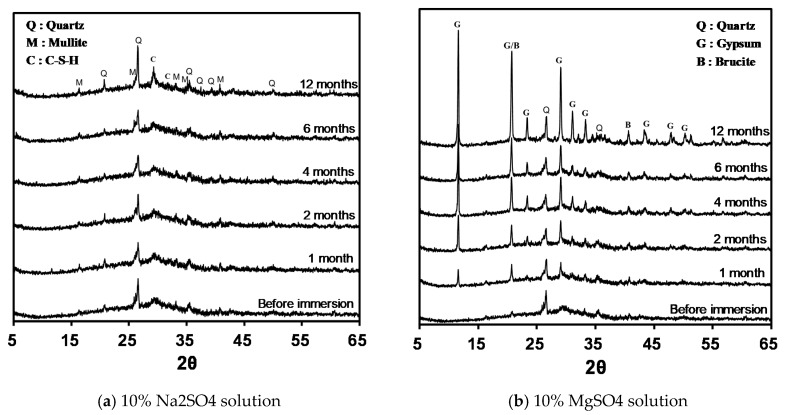
XRD patterns of S30-2.0-23 samples, immersed in (**a**) 10% Na_2_SO_4_ solution and (**b**) 10% MgSO_4_ solution.

It was confirmed through the previous experiments that the sulfate resistance of alkali-activated mortar varies depending on the type of sulfate solution applied. During this experiment, the change in mineral composition of alkali-activated paste immersed in a sulfate solution was confirmed using X-ray diffraction analysis. Figure 9a shows the results of the X-ray diffraction analysis of specimens immersed in a 10% Na_2_SO_4_ solution. No change in mineral composition was observed until after one year of immersion. Quartz (SiO_2_), mullite (3Al_2_O_3_·2SiO_2_), and C–S–H were observed as the main crystalline components of the alkali-activated GGBFS blended binder.

However, an extremely large difference can be seen from the results of the x-ray diffraction patterns in the immersion of the 10% MgSO_4_ solution, as shown in Figure 9b. Peaks of gypsum (CaSO_4_·2H_2_O) and brucite [Mg(OH)_2_] were observed from 1 month after immersion in the 10% MgSO_4_ solution, which is confirmed through the reaction of Equation (2). It is thought that the peaks of gypsum and brucite become higher as the immersion time increases owing to the increase in the amount of their production. Based on an X-ray diffraction analysis, Ismail et al. [39] reported that the deterioration by magnesium sulfate is due to the decomposition of calcium rich gel magnesium ions and the formation of gypsum. In their study, the formation of brucite and gypsum was also confirmed through the X-ray diffraction analysis. Rasheeduzzafar et al. [34] reported that the deterioration mechanism of OPC by magnesium sulfate is due to the decomposition of C–S–H by magnesium ions and the formation of gypsum and magnesium hydroxide. The formation of brucite shows that the deterioration of an alkali-activated FA/GGBFS blended mortar by magnesium sulfate proceeds in the same manner as that of OPC.

3MgSO_4_ + 3CaO·2SiO_2_·3H_2_O + 8H_2_O → 3(CaSO_4_·2H_2_O) + 3Mg(OH)_2_ + 2SiO_2_·H_2_O (5)

When an alkali-activated mortar was immersed in a 10% MgSO_4_ solution, the increases in mass and length and the decrease in the compressive strength were caused by the formation of gypsum and brucite through the reaction of magnesium ions, as shown in Equation (5) [33]. As the experiment results indicate, it can be concluded that the alkali-activated GGBFS blended mortar does not deteriorate in the presence of sulfate only, but deteriorates through the formation of gypsum and brucite when magnesium and sulfate ions are present together. In addition, FA-based geopolymers produce aluminosilicate gels with different properties from those of Ca(OH)_2_ and C–S–H through alkaline activation [52]. Because they are unaffected by magnesium ions, they show an excellent sulfate resistance regardless of the types of sulfate solution applied.

## 4. Conclusions

The following results were obtained from the evaluation of the sulfate resistance of an alkali-activated FA-based geopolymer and GGBFS blended mortar according to the type and concentration of sulfate solution, GGBFS/FA ratio, initial curing temperature, and Ms value of activator. 

(1) Alkali-activated GGBFS blended mortars, unlike FA-based geopolymer mortars, cause a significant increase in mass when immersed in a 10% MgSO_4_ solution.

(2) The compressive strength of alkali-activated mortars showed a significant variation according to the cation accompanying the sulfate. During immersion in a 10% Na_2_SO_4_ solution, the compressive strength does not decrease in all alkali-activated mortars. However, during the immersion in a 10% MgSO_4_ solution, the alkali-activated, GGBFS blended mortars show a significant decrease in compressive strength. 

(3) Alkali-activated mortars were demonstrated to be less expandable than OPC mortar immersed in a 10% Na_2_SO_4_ solution, whereas alkali-activated GGBFS blended mortars show a remarkable expansion when immersed in a 10% MgSO_4_ solution. 

(4) The value of the Ms plays an important role in the compressive strength of alkali-activated mortars, but does not affect the sulfate resistance of alkali-activated GGBFS blended mortars.

(5) The XRD results indicate that the deterioration of sulfate in an alkali-activated GGBFS blended paste is due to the decomposition of C–S–H by magnesium ions and the formation of gypsum and brucite. In addition, an alkali-activated FA-based geopolymer paste exhibits an excellent sulfate resistance regardless of the sulfate solution types because no reaction product such as Ca(OH)_2_ or C–S–H occurs.

## Figures and Tables

**Table 1:** Chemical compositions and physical properties of alkali-activated materials (FA and GGBFS).

	Chemical Compositions (mass %)									Physical Properties	
	SiO_2_	Al_2_O_3_	Fe_2_O_3_	CaO	MgO	K_2_O	Na_2_O	SO_3_	LOI	Density (kg/m^3^)	Blaine (m^2^/kg)
FA	58.9	20.9	5.30	3.80	1.31	0.74	1.69	0.50	4.87	2210	381
GGBFS	34.3	14.2	0.47	43.0	2.71	0.50	0.20	3.64	0.01	2890	428

**Table 2:** Mixture proportions of tested mortars. Note that Ms indicates the SiO_2_/Na_2_O molar ratio.

Mix	Factors				Mass Proportions (g)					
	FA (%)	GGBFS (%)	Na_2_O (%)	Ms	FA	GGBFS	NaOH	Sodium Silicate Solution	Water	Sand
FA100-1.4	100	-	8	1.4	450	-	25.4	175.8	114.4	1350
S30-2.0	70	30	4	2.0	315	135	8.2	125.6	146.0	1350
S50-1.0	50	50	4	1.0	225	225	15.7	62.8	185.5	1350
S50-1.5	50	50	4	1.5	225	225	11.9	94.2	165.8	1350
S50-2.0	50	50	4	2.0	225	225	8.2	125.6	146.0	1350

## Supplementary Materials

The following are available online at https://www.mdpi.com/1996-1944/12/21/3547/s1, Figure S1: Mass change of S30-2.0 series initially cured at 70 °C depending on sulfate solution type; Figure S2: Change in compressive strength of S30-2.0-70 samples based on sulfate solution type.
