# Supplementary material for: Importance of Cation Species during Sulfate Resistance Tests for Alkali-Activated FA/GGBFS Blended Mortars
Source: Materials (Basel). 2019 Oct 29;12(21):3547. doi: 10.3390/ma12213547 (PMC6861968; doi:10.3390/ma12213547)
Supplement: Supplementary file 1 [file materials-12-03547-s001.pdf]

materials

Article

Importance of Cation Species during Sulfate Resistance Tests for Alkali-Activated FA/GGBFS Blended Mortars

Youngkeun Cho [1], Joo Hyung Kim [2], Sanghwa Jung [3], Yoonseok Chung [2] and Yeonung Jeong [2,*]

1 Jeonnam and Jeju Branch, Korea Conformity Laboratories (KCL), 64, Oemori-gil, Yeosu-si, Jellanam-do 59631, Korea; young@kcl.re.kr
2 Construction Technology Research Center, Korea Conformity Laboratories (KCL), 199, Gasan Digital 1-ro, Geumcheon-gu, Seoul 08503, Korea; kjhmole@kcl.re.kr (J.H.K.); yschung24@kcl.re.kr (Y.C.)
3 Yeongnam Division, Korea Conformity Laboratories (KCL), 36, Technosunhwan-ro 12-gil, Yuga-eup, Dalseong-gun, Daegu 42994, Korea; jsh2593@kcl.re.kr
* Correspondence: yeonungjeong@kcl.re.kr; Tel.: +82-2-2102-2756; Fax: +82-2-856-4785

Supplementary Materials

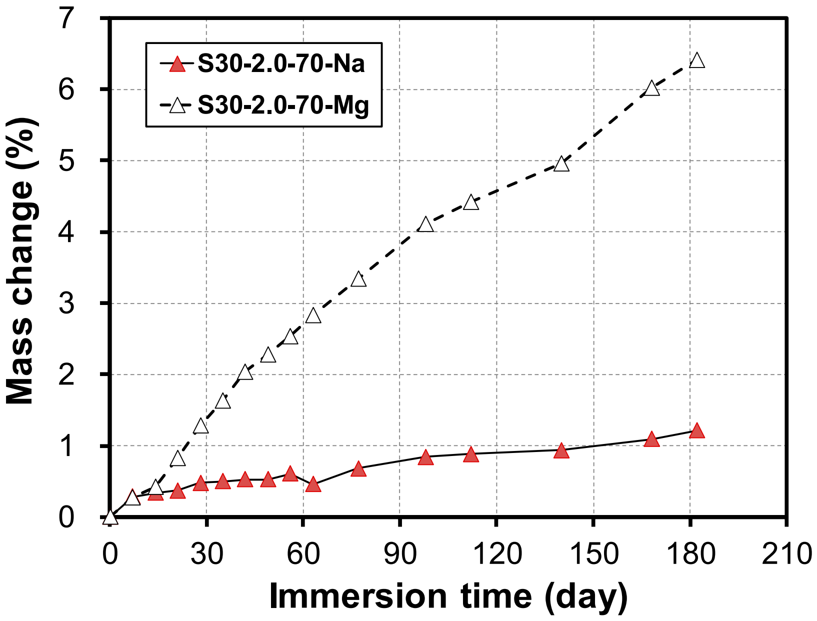

Figure S1. Mass change of S30-2.0 series initially cured at 70 °C depending on sulfate solution type.

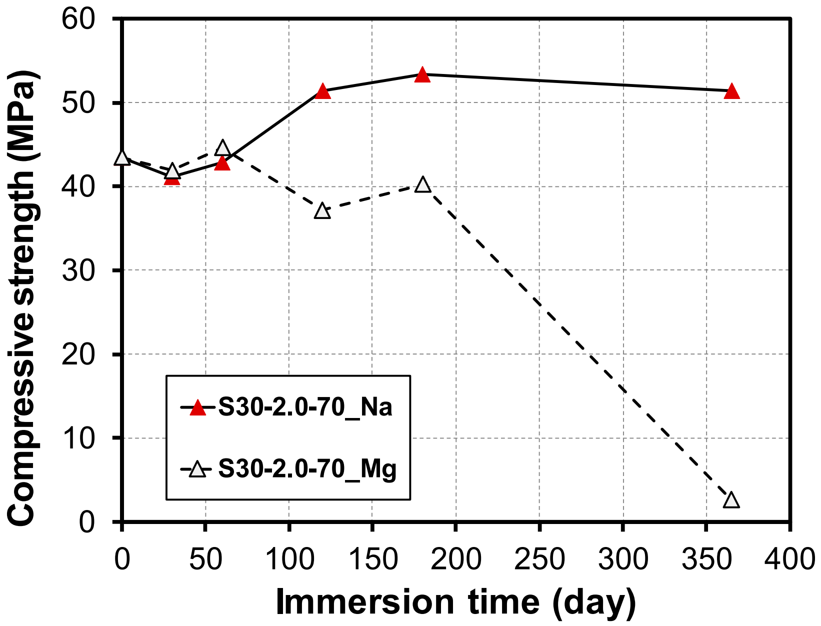

Figure S2. Change in compressive strength of S30-2.0-70 samples based on sulfate solution type.
